# Can we use short rotation coppice poplar for sugar based biorefinery feedstock? Bioconversion of 2-year-old poplar grown as short rotation coppice

**DOI:** 10.1186/s13068-017-0829-6

**Published:** 2017-06-05

**Authors:** Chang Dou, Wilian F. Marcondes, Jessica E. Djaja, Renata Bura, Rick Gustafson

**Affiliations:** 1 Biofuels and Bioproducts Laboratory, School of Environmental and Forest Sciences, University of Washington, Seattle, WA 98115 USA; 2 Department of Biotechnology, Lorena School of Engineering, University of São Paulo, Lorena, Brazil; 3 School of Environmental and Forest Sciences, University of Washington, Box 352100, Seattle, WA 98195-2100 USA

**Keywords:** Poplar, Short rotation coppice, Steam explosion, Sugar yield, Saccharification, Leaf removal, Whole tree harvest, Economic analysis

## Abstract

**Background:**

Feedstock cost is a substantial barrier to the commercialization of lignocellulosic biorefineries. Poplar grown using a short rotation coppice (SRC) system has the potential to provide a low-cost feedstock and economically viable sugar yields for fuels and chemicals production. In the coppice management regime, poplars are harvested after 2 years’ growth to develop the root system and establish the trees. The biomass from these 2-year-old trees is very heterogeneous, and includes components of leaf, bark, branch, and wood chip. This material is quite different than the samples that have been used in most poplar bioconversion research, which come from mature trees of short rotation forestry (SRF) plantations. If the coppice management regime is to be used, it is important that feedstock growers maximize their revenue from this initial harvest, but the heterogeneous nature of the biomass may be challenging for bioconversion. This work evaluates bioconversion of 2-year-old poplar coppice and compares its performance to whitewood chips from 12-year-old poplar.

**Results:**

The 2-year-old whole tree coppice (WTC) is comprised of 37% leaf, 9% bark, 12% branch, and 42% wood chip. As expected, the chemical compositions of each component were markedly different. The leaf has a low sugar content but is high in phenolics, ash, and extractives. By removing the leaves, the sugar content of the biomass increased significantly, while the phenolic, ash, and extractives contents decreased. Leaf removal improved monomeric sugar yield by 147 kg/tonne of biomass following steam pretreatment and enzymatic hydrolysis. Bioconversion of the no-leaf coppice (NLC) achieved a 67% overall sugar recovery, showing no significant difference to mature whitewood from forestry plantation (WWF, 71%). The overall sugar yield of NLC was 135 kg/tonne less than that of WWF, due to the low inherent sugar content in original biomass. An economic analysis shows the minimum ethanol selling price required to cover the operating cost of NLC bioconversion was $1.69/gallon.

**Conclusions:**

Leaf removal resulted in significant improvement in overall monomeric sugar production from SRC biomass. Leaf removal is essential to achieve good yields in bioconversion of poplar. Economic analysis suggests the NLC could be a reasonable feedstock provided it can be obtained at a discounted price.

**Electronic supplementary material:**

The online version of this article (doi:10.1186/s13068-017-0829-6) contains supplementary material, which is available to authorized users.

## Background

Biorefineries require low-cost feedstock and cost-competitive conversion processes to be economically viable. Significant progress has been made to improve biomass to fuel conversion, but feedstock cost is still a major factor impeding biorefinery commercialization [1]. For example, raw feedstocks are reported to contribute over 40% of the operating costs in lignocellulosic biomass-based ethanol production [1, 2].

Poplar, woody biomass from different species (or hybrids) within the genus *Populus*, is well recognized as an excellent bioenergy feedstock [3]. Poplar is one of the most productive temperate wood species with rapid growth rate and abundant biomass accumulation [3]. Besides growing on forest lands or farm lands, poplar can thrive on marginal lands with little fertilization or irrigation [4]. Given the diverse genetic base and available genome sequence [5], it is easy to tailor the characteristics of poplar through crossbreeding and/or genetic modification. Progresses have been made to achieve higher saccharification or fermentation yields from poplar by increasing cellulose content, altering the content and syringyl/guaiacyl (S/G) ratio of lignin, and suppressing the synthesis of certain hemicellulose [3]. As a perennial woody biomass, poplar can be harvested any time to fulfill the feedstock requirements of a biorefinery. On-demand harvest reduces feedstock handling costs by eliminating the need for feedstock storage infrastructure and avoiding degradation during long-term storage [6, 7]. In addition to these economic advantages, poplar has important traits for sustainability and possesses proven environmental benefits. Poplar tree farms provide many environmental services including carbon sequestration, erosion control, soil remediation, wastewater treatment, and wildlife habitat [8].

**Fig. 1:**
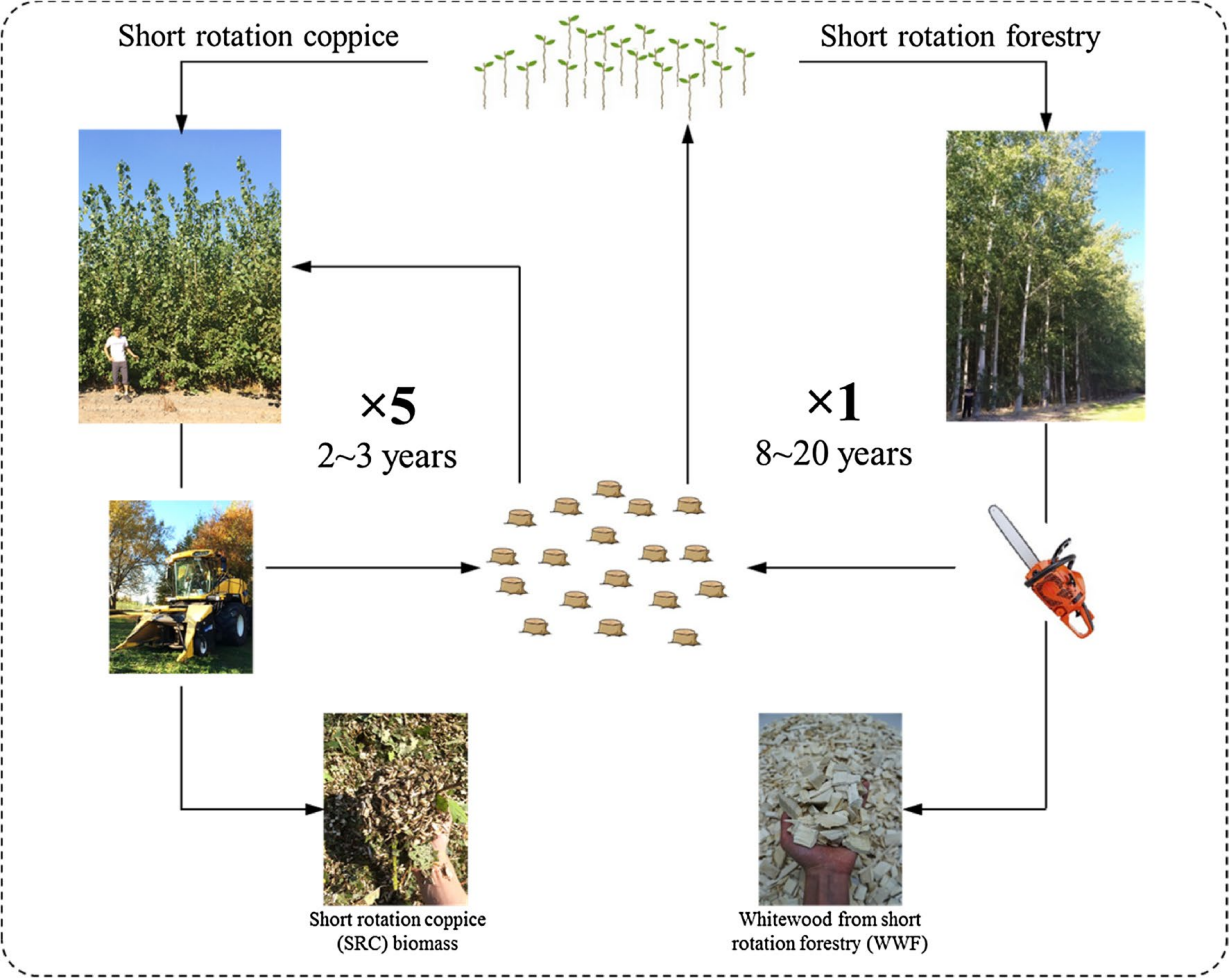
Plantation management strategies of short rotation coppice (SRC) and short rotation forest (SRF) systems

Poplar, depending on the final application, is cultivated in two types of plantation systems—short rotation coppice (SRC) and short rotation forestry (SRF) (Fig. 1). Both systems share traits of fast growing trees, ease of propagation, marginal land usage, and environmental services, but are different in terms of stand density, growing rotation, and harvest strategy (Table 1).

**Table 1:** Management strategies, current applications of poplar from short rotation forestry system (SRF), and short rotation coppice system (SRC)

	Short rotation coppice (SRC)	Short rotation forestry (SRF)
Management strategies		
Tree density (stand/ha)	≥1500 [12]	500–1500 [11, 12]
Plantation rotation (years)	2–5 [15]	8–20 [11, 13]
Productivity (odt^d^/ha year)	Up to 25 [10]^a^	6.1–16.3 [14]^b^
Harvest biomass (%)	~100	30 [16]
Applications	Heat and power	Pulpwood, sawlog, and lumber
Price ($/odt)	N/A^c^	50–120 [17]

^a^Leafless total coppice data

^b^Saw log component data

^c^Feedstock market price of SRC not available

^d^“odt” stands for oven dry tonne

The short rotation coppice (SRC) system was initially developed for energy crop production, especially solid fuel for heat and power generation [9]. Poplars are planted at densities higher than 1500 trees/ha in the SRC system and maintained using intensive agricultural-type crop management (Table 1) to achieve maximal biomass yields—up to 25 odt/ha year (research plot records) [10–12]. The high yield and whole tree harvesting result in a lower feedstock cost.

Short rotation forestry (SRF) is another tree plantation system, which has been commercialized to provide woody commodities such as pulpwood, sawlogs, and lumber to various markets. In the SRF system, poplars are managed with more conventional tree farming practices using 8–20 year rotations [11, 13] (Fig. 1). Poplars are grown as large, single-trunk trees at densities of 500–1500 trees/ha [11]. The reported productivity of SRF system is lower (6.1–16.3 odt/ha year) [14], but the products such as sawlog, lumber, and pulpwood are more valuable compared to the coppice products from the SRC system (Table 1). Poplars managed in SRC tree farms are attractive alternatives to conventional SRF practice to provide less expensive biomass for biorefineries. SRC management accelerates plant growth and maximizes land productivity, and provides revenue in as short as 2 years. Coppicing eliminates the costs of land preparation and replantation for up to 20 years (Fig. 1). Initially planted from cuttings, poplars are grown for 2 years before the first coppice to properly establish the root system. The already developed root system stimulates the growth of multiple stems per stump, supporting greater biomass accumulation in a shorter time commitment [18, 19]. In the succeeding rotations, coppicing triggers vigorous juvenile growth, accelerates plant intrinsic regeneration, and ultimately maximizes the biomass yield [9, 20]. When establishing the SRC plantation, the first cycle (a 2-year cycle) grows from the cuttings and yields less biomass per hectare compared to the subsequent rotations. However, as mentioned above, the first coppice is essential for the following rotations and is unavoidable in an approximate 20-year lifespan of SRC tree farms. The SRC poplar used in this study is originated from the first cycle after 2-year growth.

Harvesting represents one of the most energy-intensive operations in the tree farm practice and adds a significant cost in the overall feedstock supply chain [21]. Small-dimensioned trees in SRC system (2–5 years depending on the growth) can be harvested efficiently using fully mechanized agricultural machinery which integrates cutting and chipping. Schweier et al. [22] evaluated a “trees to chips” harvester and described the system as reliable and cost-efficient.

Bioconversion of debarked poplar wood from mature trees has been extensively studied [23]. In addition, there has been some research on bioconversion of poplar with bark to assess how heterogeneous woody biomass might perform. Those investigations gave mixed results with DeMartini [24] reporting that bark was inhibitory to enzymatic actions, while Schütt [25] found no significant difference between debarked and non-debarked poplar during enzymatic hydrolysis of steam-pretreated solids. No one has investigated bioconversion of whole tree poplar, which contains (whitewood) chips, bark, branches, and even leaves if harvest is done in the growing season. In addition, the material that will be produced in the initial 2-year harvest is very heterogeneous. Poplar for biofuels will be grown using the SRC system because of its earlier growth culmination and shorter harvest cycle. It is essential, therefore, that the performance of this material for bioconversion be thoroughly assessed. The purpose of our research is to make this assessment. This paper presents research on the bioconversion of SRC poplar from the initial 2-year harvest. It aims to 1) characterize the SRC poplar in terms of physical and chemical properties, 2) investigate and improve the sugar production of SRC poplar in bioconversion, and 3) evaluate the economics of SRC poplar to biofuel process. Subsequent papers will present work on bioconversion of poplar coppice harvested in ensuing rotations.

## Methods

**Fig. 2:**
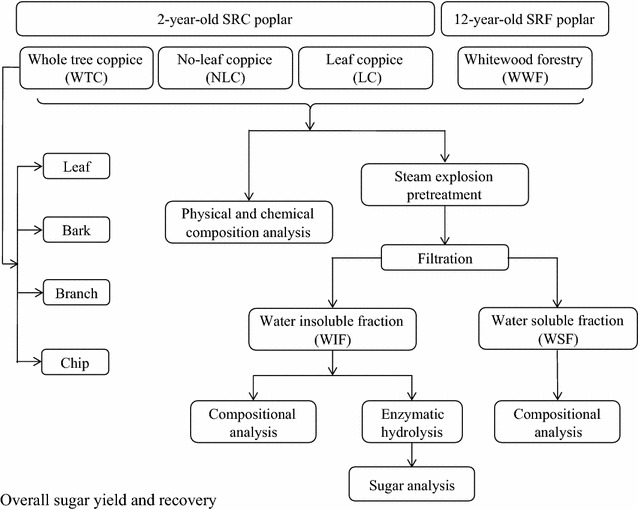
Process flow diagram for bioconversion of different poplar feedstocks (WTC, NLC, LC, and WWF) into sugars

SRC poplars were harvested and chipped using a whole tree harvester after 2-year growth following planting. The harvested biomass, the whole tree coppice (WTC), was characterized by separating and analyzing its four main components: leaf, bark, branch, and (whitewood) chip. By sorting out leaves, we obtained leaf coppice (LC, including only leaf) and no-leaf coppice (NLC, including bark, branch, and chip) (Fig. 2). The chemical compositions of WTC, NLC, and LC were analyzed and compared with whitewood from forestry (WWF). The four poplar feedstocks were then processed in the same way through pretreatment and enzymatic hydrolysis. The WTC, NLC, LC, and WWF were steam-pretreated at 195 °C for 5 min with SO_2_ (3% w/w) impregnation. After separation, the chemical compositions of the water-insoluble fractions (WIF) and water-soluble fractions (WSF) were analyzed. Water-insoluble fractions were then enzymatically hydrolyzed at 5% (w/v) consistency and 5 FPU/g cellulose enzymes loading (Fig. 2). The overall monomeric sugar yields (kg monomeric sugars/tonne biomass) and recoveries (kg monomeric sugars/kg original sugars × 100%) were used to assess the impact of leaf removal following steam pretreatment and enzymatic hydrolysis, and to evaluate the efficiency of using poplar coppice in bioconversion. The experimental results were input into a modified NREL biochemical conversion model for economic analysis [1].

### Raw material

Two-year-old SRC poplar used in this research is a hybrid of *Populus trichocarpa* and *Populus deltoides*, obtained from a plantation near Jefferson, OR managed by GreenWood Resources (Portland, OR). In spring 2012, poplars were established with 10″ to 22″ long cuttings in rows with at high density of 1452 stems per acre (3630 stems per hectare). In September 2013, the harvest of stock like crops occurred before defoliation. The 2-year-old poplar was harvested and chipped using a fully mechanized harvester—a modified forage harvester New Holland FR9080 (New Turin, Italy) equipped with 130FB biomass header [22] (Fig. 1). As Fig. 2 shows, NLC and LC were prepared by separating the leaf component from 5 kg original coppice feedstock. One kg (OD) of the WTC was manually sorted into four components: leaf, bark, branch, and (whitewood) chip. After sorting, the individual components were oven-dried to determine the physical composition of the feedstock. In the current study, all the biomass obtained from the 1st cycle harvest are identified as coppice samples. Chips from mature 12-year-old poplars (*Populus deltoides* × *Populus nigra,* clone OP-367), also provided by GreenWood Resources, were included in this study as reference biomass. Fresh poplar was debarked and chipped into 1.0 cm × 2.0 cm at 5 mm thickness chips. These clean poplar chips are termed whitewood forestry (WWF). All samples were stored and kept frozen at −20 °C until use.

### Pretreatment and processing conditions

Four types of poplar feedstocks, WTC, NLC, LC, and WWF, were processed in the same condition. Each feedstock of 600 g oven-dried (OD) weight was pre-impregnated overnight with anhydrous SO_2_ in plastic bags at atmospheric pressure. The amount of SO_2_ added to the bag corresponded to 3% (w/w) loading, and was determined by weighing the bag before and after the addition of gas.

Steam explosion pretreatment was performed in a 2.7 L batch reactor (Aurora Technical, Savona, BC, Canada). Briefly, samples were loaded and heated at temperature 195 °C for 5 min. Following the reaction time, the pneumatic valve was opened to explode and discharge the biomass into a collection container.

After steam explosion, the pretreated biomass slurry was separated into WSF and WIF using vacuum filtration. The WIF was then washed with a volume of deionized water equivalent to 20 times the dry weight of the sample to remove the free sugars.

### Enzymatic hydrolysis

Enzymatic hydrolysis was carried out using cellulase (Celluclast 1.5 l, Sigma) at 5 Filter Paper Units (FPU)/g cellulose and *β*-glucosidase (Novozyme 188, Sigma) at 10 cellobiase units (CBU)/g cellulose. The WIF was hydrolyzed at 5% (w/v) consistency in a total volume of 50 ml in 125 ml Erlenmeyer flasks. The flasks were incubated at 50 °C and 175 rpm in a New Brunswick shaker. Additionally, 50 mM citrate buffer was added to maintain the pH at 4.8, and tetracycline (40 µg/ml) and cycloheximide (30 µg/ml) were used to inhibit microbial contamination. One ml samples were taken periodically, boiled for 10 min to denature enzymes, filtered through a 0.22 µm syringe filter, and stored at −20 °C until analysis.

### Compositional analysis

#### Ash and extractives

Ash content of raw biomass samples was measured gravimetrically by heating 20-mesh-milled dry biomass to 575 ± 25 °C for 18 ± 6 h [26]. Water and ethanol extractives of raw biomass were determined according to National Renewable Energy Laboratory (NREL) methods [27].

#### Soluble fraction carbohydrates and degradation products

Monomeric/oligomeric soluble carbohydrates and degradation products were determined using NREL LAP TP-510-42623 [28]. Briefly, 0.7 ml of 72% H_2_SO_4_ was added to 15 ml of the liquid samples, and the volume made up to 20 ml with water. Samples were autoclaved at 121 °C for 60 min. The samples were then analyzed by a Dionex (Sunnyvale, CA) HPLC (ICS-3000) system equipped with an anion exchange column (Dionex, CarboPac PA1) and an electrochemical detector using deionized water at a flow rate of 1 ml/min as an eluent [29]. Oligomeric sugar was calculated by subtracting monomeric sugar content from total sugar content determined after acid hydrolysis.

Degradation products, such as acetic acid, furfural, and HMF, were determined using a Shimadzu Prominence LC equipped with an anion exchange column (Rezex RHM Monosaccharide H^+^ (8%) Phenomenex, Inc., Torrance, CA) using 5 mM H_2_SO_4_ at 0.6 ml/min [29]. Phenolic concentration in the WSF was assayed by the Folin Ciocalteu method [30] using a spectrophotometer (Shimadzu, Tokyo, Japan) at 765 nm. Gallic acid was used as calibration standard.

#### Insoluble fraction carbohydrates, acetate groups, and phenolics

The chemical compositions of raw biomass and WIF were determined according to a modified method derived from TAPPI Standard Method [31] and NREL protocols [32]. Briefly, 0.2 g of finely ground oven-dried sample was treated with 3 ml 72% H_2_SO_4_ for 120 min at room temperature, and then diluted into 120 ml total volume and autoclaved at 121 °C for 60 min. Klason lignin contents were determined by gravimetric methods to estimate phenolic content. It should be noted that in components such as leaf, branch, and bark, phenolic compounds are not necessarily fully lignified and therefore are defined as phenolics other than lignin [33]. After filtration through tared sintered glass crucibles, the carbohydrate and acetyl compositions of the filtrate are analyzed by HPLC, and the acid soluble lignin (phenolics) in the filtrate is analyzed by UV at 205 nm [29].

### Sugar yield and recovery calculation

A complete mass balance was calculated using the composition and total mass of each WSF and WIF leaving pretreatment and enzymatic hydrolysis [23]. Yields and recoveries were calculated based on the input feedstock mass and original sugars available in the raw feed, respectively. In this manuscript, “yield” was defined as the amount of sugars converted from unit weight of feedstock. It is determined based on the total mass of sugars in the solid and liquid phases normalized by the initial oven dry mass of biomass (kg sugars/tonne biomass). Recovery is defined as the percentage of theoretical sugar yield by calculating the total mass of sugars in the solid and liquid phases normalized by the initial mass of sugars in the biomass (kg sugars/kg original sugars × 100%). Similarly, monomeric sugar yield is defined as total mass of monomeric sugars in the hydrolyzed solid and liquid phases normalized by the initial oven dry mass of biomass (kg monomeric sugars/tonne biomass), and monomeric sugar recovery is defined as the total mass of monomeric sugars in the hydrolyzed solid and liquid phases normalized by the initial mass of sugars in the biomass (kg monomeric sugars/kg original sugar × 100%).

### Buffering capacity test

The buffering capacity of SRC biomass was investigated by titration as described by [34]. Briefly, 50 g OD weight of raw biomass was soaked in 1 l deionized water at a temperature of 80 °C for 30 min. Biomass was then removed by filtration and 800 ml of liquid was titrated by 0.1 M H_2_SO_4_ to a pH of 1.5. Deionized water was used as blank for reference.

### Economic assessment

The economic potential for using the 2-year 1st cycle coppice as a biorefinery feedstock was assessed. It was assumed that this material is an intermittent feedstock and the biorefinery would be designed to handle the feedstock harvested from subsequent rotations. We determined the ethanol selling price the biorefinery would need to just cover the operating cost of producing alcohol from the 2-year-old coppice. Any selling price less than this would result in the biorefinery losing money and would not be an acceptable. The minimum ethanol price was calculated following the method outlined in the 2011 biochemical conversion of lignocellulosic ethanol report from NREL [1], modified for the poplar feedstock with the cost updated to 2015 USD. An Aspen Plus model (Aspen Plus™, V8.6) of the biorefinery was developed using the yields and recoveries determined in this research. Process yields are the most critical variable driving process economics and the process yields used in the Aspen model are given in the Additional file 1: Table S1. The feedstock price was determined from the heating value of the poplar with the assumption that the most realistic market for the 2-year-old trees would be hog fuel. A price of $2.8/MMBtu ($0.00265/MJ) was used for the calculation [35].

### Data analysis

The results were subjected to one-way analysis of variance (ANOVA) analysis followed by a Tukey’s test. Triplicate samples were separate runs conducted for each type of feedstock. All data are represented as the mean of triplicates with standard deviation. The percent differences in data comparison represent the absolute values, unless otherwise indicated. Chemical composition, sugar conversion of enzymatic hydrolysis, sugar yield, and recovery following steam pretreatment, and monomeric sugar yield and recovery after steam pretreatment and enzymatic hydrolysis were analyzed based on 5% alpha level (95% confidence interval). Statistical differences in chemical composition, physical characteristics, and sugar yield were determined from *p* values (*p* < 0.05). Data were analyzed using R (version 3.0.1) software. In this manuscript, any data analysis mentioned as “significant” represents statistically significant (*p* < 0.05).

## Results and discussion

### Physical and chemical compositions of SRC biomass

As Table 2 shows, WTC consisted of 37% leaf, 9% bark, 12% branch, and 42% chip. Chips comprised less than half of the dry WTC, while leaves comprised over one-third of the total dry biomass. The relatively small chip content was mainly due to the tree age and management practice [36]. In a conventional poplar plantation, leaf, bark, and branch components are considered low value and may be discarded after harvest.

**Table 2:** Physical and chemical compositions of four components in SRC poplar and SRF poplar

	Physical composition (%)	Chemical composition (%)									
		Arabinan	Galactan	Glucan	Xylan	Mannan	Total sugar	Total phenolics	Acetic acid	Ash	Extractives
WTC											
Leaf	37	2.9 ± 0.1	2.3 ± 0.1	13.1 ± 0.6	3.9 ± 0.2	0.6 ± 0.1	22.7 ± 0.1	39.8 ± 0.1	2.3 ± 0.3	10.5 ± 0.1	27.6 ± 1.3
Bark	9	4.0 ± 0.1	2.1 ± 0.1	24.4 ± 0.1	4.1 ± 0.1	0.6 ± 0.1	35.2 ± 0.1	32.9 ± 0.3	2.6 ± 0.2	6.9 ± 0.2	27.7 ± 0.5
Branch	12	2.9 ± 0.1	1.8 ± 0.1	23.9 ± 0.1	7.2 ± 0.4	0.9 ± 0.1	36.8 ± 0.1	29.2 ± 0.4	3.4 ± 0.2	5.7 ± 0.1	21.9 ± 0.9
Chip	42	0.5 ± 0.1	0.7 ± 0.1	38.5 ± 0.3	14.3 ± 0.1	1.8 ± 0.2	55.8 ± 0.2	24.2 ± 0.2	4.4 ± 0.3	1.3 ± 0.1	12.3 ± 0.4
WWF	N/A	0.4 ± 0.0	0.4 ± 0.1	46.5 ± 0.9	13.1 ± 0.2	2.6 ± 0.1	63.0 ± 0.4	25.3 ± 0.2	1.2 ± 0.1	0.7 ± 0.1	5.2 ± 0.4

± All data are represented as the mean of triplicates with standard deviation

The chemical compositions of the four main components are listed in Table 2. It was found that the chemical compositions across the four main components were significantly different. Among all components, chip represented the best general chemical composition for biofuel production, with the highest sugar content (55.8%), the lowest total phenolic content (24.2%), and just 1.3% ash. In contrast, leaf had the lowest total sugar (22.7%) but the highest total phenolic and ash contents (39.8% and 10.5%, respectively). In bark and branch, the total sugar, total phenolic, and ash contents were in between those of chip and leaf.

As Table 2 reveals, the WWF exhibited significantly higher total sugar content, and lower acetic acid, ash, and extractives contents than all the components from WTC. Although the chip was most suitable for bioconversion among four WTC components, it was still less desirable than WWF. In particular, WWF had a 7.2% higher total sugar content, 0.7% lower ash content, and 7.1% less extractives compared to the chip from WTC. Previous studies demonstrated that the radius, length, and cell wall thickness of fibers increase as tree ages, and as a result the chemical composition differs between juvenile and mature wood [37]. Our observations confirmed the fact that juvenile wood is lower in cellulose, but higher in lignin, ash, and extractives than mature wood [24, 38].

The low sugar content leaf consisted of 37% of the total dry WTC biomass (Table 2). Consequently, the presence of leaf increased the non-carbohydrate constituent and reduced the proportion of convertible sugar in WTC. Additionally, high phenolic content, ash, and extractives in the leaves will have negative impacts on the bioconversion process [39–42]. The leaves appeared to be problematic. So we conducted a leaf separation to change the raw biomass chemical composition and examine the impacts of leaf removal in bioconversion.

Table 3 presents the compositional analysis of NLC, WTC, LC, and WWF. Leaf removal significantly changed the physical composition of SRC biomass, increasing chip composition to 67%. The original biomass, WTC, was composed of 41.3% total sugar, 32.1% total phenolics, 3.7% acetic acid, 5.5% ash, and 21.0% extractives (Table 3). Besides glucan and xylan, the WTC consisted of 5.0% of minor sugars, which were mainly attributed to the presence of bark, branch, and leaf, as these sugars are rarely observed in mature poplar wood [37]. Following leaf removal, the sugar content increased by 8.2%, while total phenolic, ash, and extractives contents decreased by 5.3, 2.1, and 4.3%, respectively. Leaf removal generated a feedstock (NLC) with a 49.5% total sugar content. The total sugar content of the NLC was still 13.6% lower, and the ash and extractives contents were 4 and 2 times higher, respectively, compared to WWF.

**Table 3:** Chemical and physical compositions of NLC, WTC, LC, and WWF

	Physical composition (%)				Chemical composition (%)									
	Leaf	Bark	Branch	Chip	Arabinan	Galactan	Glucan	Xylan	Mannan	Total sugar	Total phenolics	Acetic acid	Ash	Extractives
NLC	0	15	18	67	1.5 ± 0.1	1.3 ± 0.1	33.2 ± 0.5	11.9 ± 0.3	1.6 ± 0.1	49.5 ± 0.2	26.8 ± 1.0	4.3 ± 0.3	3.4 ± 0.0	16.7 ± 0.0
WTC	37	9	12	42	2.1 ± 0.1	1.7 ± 0.1	26.9 ± 0.5	9.4 ± 0.3	1.2 ± 0.1	41.3 ± 0.2	32.1 ± 1.0	3.7 ± 0.1	5.5 ± 0.1	21.0 ± 0.6
LC	100	0	0	0	2.9 ± 0.1	2.3 ± 0.1	13.1 ± 0.6	3.9 ± 0.2	0.6 ± 0.0	22.7 ± 0.1	39.8 ± 0.1	2.6 ± 0.5	10.5 ± 0.1	27.6 ± 1.3
WWF	0	0	0	100	0.4 ± 0.0	0.4 ± 0.1	46.5 ± 0.9	13.1 ± 0.2	2.6 ± 0.1	63.1 ± 0.4	25.3 ± 0.2	1.2 ± 0.1	0.7 ± 0.1	5.2 ± 0.4

± All data are represented as the mean of triplicates with standard deviation

### Chemical composition of water-insoluble fraction (WIF) after pretreatment

Following pretreatment and liquid–solid separation of all samples, the compositions of the solid, water-insoluble fraction (WIF), and the liquid, water-soluble fraction (WSF), were analyzed. Expressed as percent of dry matter, Table 4 shows that the trends of the WIF chemical constituents were generally consistent with the compositional data in the raw biomass. The sugar and phenolic contents of three coppice samples ranged from 17.9% to 55.2% and from 37.4% to 50.5%, respectively. Comparing WTC and NLC shows that leaf removal increased the WIF sugar content by 13.8% and lowered the phenolic and ash contents by 6.2% and 3.8%, respectively. Typically, WIF of LC contained the lowest sugar content, but the highest phenolic and ash contents. Although leaf removal significantly enhanced the sugar content and reduced the phenolic and ash contents of WIF, the NLC sugar content was still 8.7% lower, and phenolics and ash were 6.2% and 1.9%, respectively, higher than WWF.

**Table 4:** Chemical composition of WIF after steam pretreatment of poplar samples (as percentages of the solid weight)

	Chemical composition (%)								
	Arabinan	Galactan	Glucan	Xylan	Mannan	Total sugar	Total phenolics	Acetic acid	Ash
NLC	0.1 ± 0.1	0.1 ± 0.1	52.2 ± 0.3	2.4 ± 0.3	0.5 ± 0.1	55.2 ± 0.1	37.4 ± 0.1	1.6 ± 0.2	2.0 ± 0.1
WTC	0.1 ± 0.1	0.4 ± 0.1	36.6 ± 0.1	3.6 ± 0.3	0.7 ± 0.1	41.4 ± 0.1	43.6 ± 0.1	1.9 ± 0.1	5.8 ± 0.3
LC	0.4 ± 0.1	0.9 ± 0.2	13.1 ± 2.9	3.2 ± 0.6	0.4 ± 0.1	17.9 ± 0.6	50.5 ± 0.2	2.5 ± 0.9	9.8 ± 0.4
WWF	0.0 ± 0.0	0.0 ± 0.0	62.4 ± 2.4	1.1 ± 0.1	0.4 ± 0.1	63.9 ± 0.3	31.2 ± 0.1	0.0 ± 0.0	0.1 ± 0.1

± All data are represented as the mean of triplicates with standard deviation

### Chemical composition of water-soluble fraction (WSF) after pretreatment

The amount of sugars, degradation products, and the pH in the WSF after pretreatment were measured. Table 5 shows that sugar yields in the WSF varied across different poplar samples. Consistent with the results in Table 4, the majority of minor sugars resided in the WSF, as hemicellulose was mostly dissolved during pretreatment for all poplar samples. Sugar yields are presented in the unit of kg/tonne, implying the total soluble sugars released during pretreatment. For the coppice samples, the glucose and xylose yields ranged between 15.8 to 63.5 and 6.9 to 96.0 kg/tonne, respectively. Minor sugars, including arabinose, galactose, and mannose, represented a non-negligible composition in the WSF, and their yields ranged between 7.6–9.7, 5.6–8.5, and 1.1–14.3 kg/tonne, respectively. The trends of total sugars in WSF matched the sugar content in original biomass. As the amount of the individual sugars decreased from NLC to LC (Table 5), the total sugars decreased from 192.1 to 37.0 kg/tonne in the WSF. The monomeric sugar percentage in the WSF is critical because it indicates the amount of direct fermentable sugars. Most sugars in the WSF were recovered in monomeric form except for the LC biomass. As shown in Table 5, leaf removal significantly changed the total amount of dissolved sugar in WSF—from 108 kg/tonne of WTC to 192 kg/tonne of NLC—an increase of 83.8 kg/tonne. Meanwhile, leaf removal increased the proportion of monomeric sugars by 12%. The fermentable sugar yield in WSF was markedly increased by removing leaves. The sugar yield in WSF of WWF, however, was 18.2 kg/tonne higher than NLC. Interestingly, the monomeric sugar contents of WWF and NLC were about the same at 82% and 83%, respectively.

**Table 5:** Sugar yield in WSF after steam explosion (expressed as kg/tonne raw biomass)

	Arabinose		Galactose		Glucose		Xylose		Mannose		Total sugars	
	Total	% mon^a^	Total	% mon	Total	% mon	Total	% mon	Total	% mon	Total	% mon
NLC	9.7 ± 0.3	100	8.5 ± 0.2	90	63.5 ± 2.5	78	96.0 ± 1.2	81	14.3 ± 1.1	79	192.1 ± 0.1	82
WTC	9.1 ± 1.2	100	7.5 ± 0.7	71	39.2 ± 4.1	64	45.5 ± 2.4	71	7.0 ± 0.3	65	108.3 ± 0.3	70
LC	7.6 ± 0.2	60	5.6 ± 0.8	23	15.8 ± 2.1	41	6.9 ± 1.2	10	1.1 ± 0.1	33	37.0 ± 0.2	36
WWF	2.9 ± 0.2	93	5.4 ± 0.4	79	62.9 ± 0.7	76	122.2 ± 8.9	87	16.8 ± 1.0	85	210.3 ± 0.5	83

± All data are represented as the mean of triplicate measurement

^a^“% mono” describes the percentage of soluble sugar presented in monomeric form

**Fig. 3:**
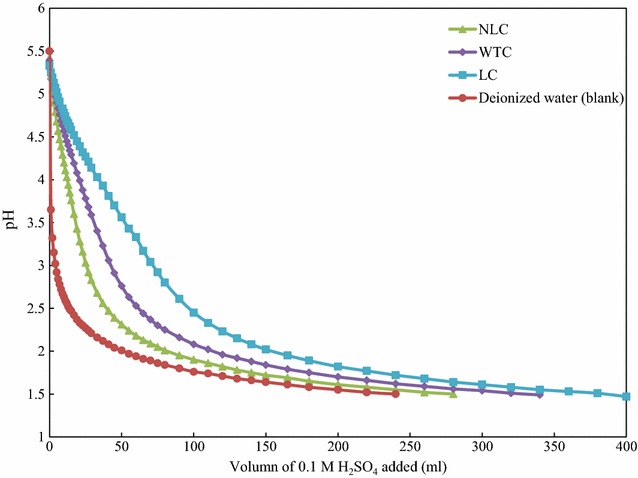
Titration curves with 0.1 M H_2_SO_4_ for water extracts of NLC, WTC, LC, and the deionized water (blank)

It is shown in Table 6 that the degradation products, including acetic acid, furfural, 5-hydroxymethyl furfural (HMF), and phenolics, were found at different amounts in the WSFs. Leaf removal increased the amount of acetic acid, furfural, and HMF in the WSF by 10.0, 8.3, and 0.4 kg/tonne, respectively (Table 6). It is known that with an increase of pretreatment severity, more sugars will be solubilized as monosaccharides and potentially more will be degraded into furans [29]. All samples were steam-pretreated at the same reaction temperature and residence time with the same SO_2_ loading, however, the pH of WSF decreased from 3.1 to 1.9 as a result of leaf removal (Table 6). The lower pH in the WSF from the NLC is partially a result of the higher acetic acid concentration compared to that from WTC. In addition, the ash in the leaf appears to provide some buffering capacity to the WSF [43]. Figure 3 shows the titration curve of three coppice samples in comparison with the blank (deionized water). It appears that for any level of acid addition, the pH of extractant from the leafy material is higher than the blank or any of the other biomass type, demonstrating the larger buffering capacity of the leaves.

**Table 6:** Acetic acid, furans, phenolics yields (expressed as kg/tonne raw biomass), and pH of WSF after steam explosion

	pH	Acetic acid	Furfural	HMF^a^	Phenolics
NLC	1.9	48.8 ± 1.5	14.8 ± 1.5	2.2 ± 0.1	23.2 ± 2.0
WTC	3.1	38.8 ± 1.3	6.5 ± 0.9	1.8 ± 0.2	21.9 ± 1.8
LC	3.7	25.2 ± 0.9	0.5 ± 0.1	0.6 ± 0.1	28.1 ± 0.2
WWF	1.6	33.5 ± 0.6	5.2 ± 0.4	1.6 ± 0.1	8.0 ± 0.4

± All data are represented as the mean of triplicate measurement

^a^5-hydroxymethyl furfural

### Sugar yield and recovery after steam pretreatment

**Fig. 4:**
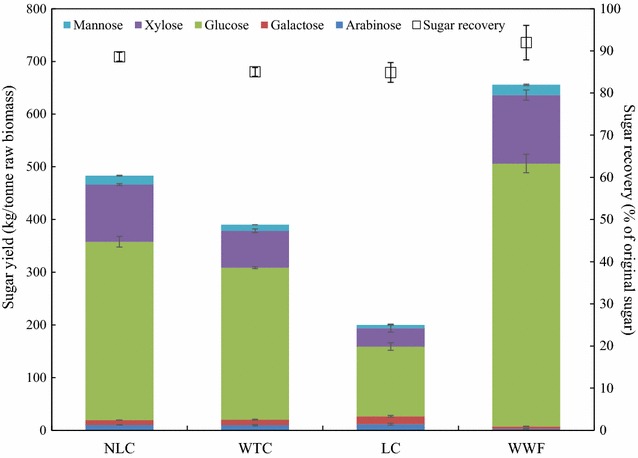
Sugar yield (kg/tonne), expressed as total mass of sugar per unit raw biomass, and sugar recovery (%), expressed as total mass of sugar per unit original sugar after pretreatment, of NLC, WTC, LC, and WWF. *Error bars* indicate standard deviation from triplicate measurements

Following steam pretreatment, total sugar yield was calculated for each of the poplar samples by combining the sugars in WIF (Table 4) and WSF (Table 5). Figure 4 presents the total sugar yield for each poplar sample. The yields ranged from 200 to 656 kg/tonne. Corresponding to the compositional characteristics of raw biomass (Table 3), NLC achieved the highest sugar yield of 483 kg/tonne among the three coppice samples, WTC had an intermediate sugar recovery of 390 kg/tonne, whereas LC had the lowest sugar recovery of 200 kg/tonne. WWF recovered 656 kg/tonne sugar in total because of its high sugar content in the original biomass. Leaf removal improved the total sugar yield by 93 kg/tonne after steam pretreatment. Interestingly, the sugar recovery was similar across all poplar samples, ranging from 85 to 92%. Although the total sugar yield was much lower in NLC, its sugar recovery showed no significant difference from WWF (Fig. 4), indicating that the large difference in sugar recoveries was mainly due to the different original sugar contents (Table 3).

### Enzymatic hydrolysis of water-insoluble fraction (WIF)

**Fig. 5:**
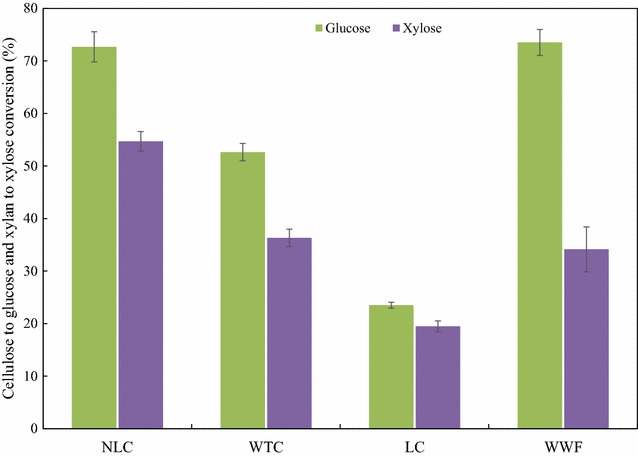
72 h cellulose to glucose and xylan to xylose conversion of steam-pretreated NLC, WTC, LC, and WWF at 5% (w/v) solids consistency with 5 FPU/g cellulose and 10 CBU/g cellulose enzyme loading. *Error bars* indicate standard deviation from triplicate measurements

Following steam pretreatment, the enzymatic digestibility of WIF samples was evaluated at 5% (w/v) consistency with 5 FPU/g cellulose enzyme loading. Figure 5 highlights the differences in digestibility between poplar samples after 72 h of saccharification. Results in Fig. 5 reveal that NLC had the highest overall sugar conversion, a 72.7% cellulose to glucose conversion, and a 54.7% xylan to xylose conversion. The WTC had lower hydrolysis conversions of 52.6% glucose conversion and 36.3% xylose conversion. Notably, leaf removal improved the enzymatic hydrolysis, resulting in 20% higher glucose conversion and 18% higher xylose conversion. LC had the lowest glucose conversion (23.5%) and xylose conversion (19.5%). By comparison, the glucose conversion of WWF (73.5%) was similar to NLC, while the xylose conversion was lower (34.1%).

The digestibility of pretreated coppice samples demonstrated an improvement in sugar conversion with leaf removal and underscored the low digestibility of steam-pretreated LC. The low yield and digestibility of the LC are attributed to the enzyme inhibition of phenolic compounds and ash. Phenolic compounds have been reported to inhibit and/or deactivate cellulase and *β*-glucosidases [41, 42]. The steam-pretreated coppice samples, especially WTC and LC, had relatively high phenolic contents of 43.6 and 50.5%, respectively (Table 4). Contrary to whitewood chips where more phenolic compounds are lignified, the foliage phenolic compounds are present in the form of low molecular weight polyphenols [33] such as tannin and some flavonoids. These low molecule polyphenols can be easily broken into monomeric phenolics during pretreatment, resulting in stronger enzyme inhibition [42]. It has also been suggested that ash in steam-pretreated biomass, especially the metal ions, hindered the action of cellulase and β-glucosidases once it exceeds certain content thresholds [40]. Compared to NLC, the two-fold greater WIF ash content of WTC could partially explain the 20% lower cellulose conversion and 18% lower xylan conversion. Leafy material with its high phenolic and ash content strongly inhibits enzymatic hydrolysis. Prior leaf removal will be necessary to achieve high saccharification yields at modest enzyme loadings.

### Overall sugar yield and recovery after steam pretreatment and enzymatic hydrolysis

**Fig. 6:**
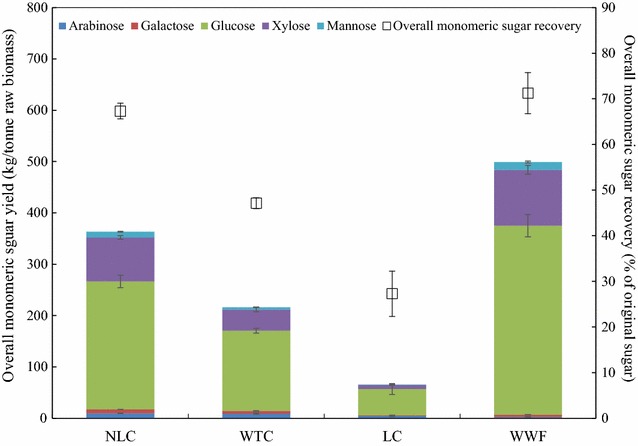
Overall monomeric sugar yield (kg/tonne), expressed as monomeric sugar per unit raw biomass, and overall monomeric sugar recovery (%), expressed as monomeric sugar per unit original sugar after pretreatment and enzymatic hydrolysis, of NLC, WTC, LC, and WWF. *Error bars* indicate standard deviation from triplicate measurements

The overall monomeric sugar available after pretreatment and enzymatic hydrolysis determines the total amount of fermentable sugars from bioconversion, and is calculated by adding monomeric sugars in WSF and hydrolyzed WIF (Fig. 6). For each biomass, bioconversion efficiency is also expressed in two formats—the overall monomeric sugar yield at kg/tonne demonstrates the monomeric sugar from per unit biomass, and overall monomeric sugar recovery (in percentage) represents the monomeric sugar from original sugar in raw biomass. Within all three coppice samples, NLC had the highest overall sugar yield of 363 kg/tonne and LC had the lowest of 66 kg/tonne. The highest overall sugar yield of NLC is a direct result of the high sugar content in original biomass (Table 3), high sugar recovery in WSF and WIF, and the most efficient WIF digestibility. Leaf removal increased the overall sugar yield by 147 kg/tonne, which accounts for 40% relative increase from the WTC (Fig. 6). Not surprisingly, only 27% original sugars were recovered from LC. NLC recovered 67% sugar after pretreatment and enzymatic hydrolysis, showing no significant difference with WWF (71%). It appears that the branch and bark in the NCL does not impair the bioconversion sugar recovery efficiency. The inherent sugar content was lower in original NLC biomass, however, resulting in 136 kg/tonne less total sugar yield of NLC compared to WWF (Fig. 6).

The differences in sugar yields for NLC and WTC show that leafy material hinders the bioconversion sugar recovery. Moreover, since leaf had an extremely high moisture content (around 70%, data not shown), the mass proportion of leaf will be even higher in fresh biomass. From a logistic point of view, the high moisture content will accelerate microbial-driven deterioration of the biomass during transportation and storage [6]. Considering the difficulty in removing leaf from the harvested poplar mixture (as we experienced), we suggest a leaf separation operation during harvest. Thompson et al. [44] have developed an air classification method to separate the heterogeneous biomass mixture into anatomical or visually unique fractions. Ideally, a similar air separation mechanism can be integrated into the coppice harvester to efficiently classify and collect both leaf and no-leaf components during harvest.

### Economic assessment

With the assumptions that NLC poplar could be purchased for its heating value—$53/tonne—we calculated the ethanol selling price required to cover the operating cost using the yield data in the current study. The simulated biorefinery used 700,000 tonne of feedstock per year and produced 144 million l/year (38 million gallon/year) at an ethanol conversion of 206 l/dry tonne feedstock (49 gallon/dry US ton feedstock). The minimum ethanol selling price required to cover the operating cost was found to be $1.69/gallon, which is approximately the same as the current ethanol selling price ($1.65/gallon, 2017). Despite the low bioconversion yield, it appears that the NLC biomass could be a reasonable feedstock provided it can be obtained at a discounted price reflecting its lower quality.

The economics of using short rotation coppice (SRC) poplars may be improved if higher value materials can be produced from leaves separated before bioconversion. In a recent review study, Devappa et al. [45] summarized a number of value-added natural chemicals which can be extracted and purified from tree residues including leaf. Tree foliage is also recognized as a high-quality supplement for animal feed [46]. Traditionally, poplar leaf has been used as a crude protein fodder resource for livestock, particularly for ruminant animals [13, 47]. In this current study, we were able to obtain about 12% of crude protein from the LC following a sonication protein extraction method (data not shown).

Returning leafy material to the tree farm could also have environmental benefits. Ecologically, foliage serves several important functions in plantation soil [48]. It improves soil structure and increases solid porosity for better aeration and moisture holding capacity. In addition, upon decomposition, foliage litter returns the minerals and organics to the site, and becomes a source of plant nutrients [48]. Complete leaf removal during whole tree harvest might increase the potential of soil erosion, site degradation, and accelerate nutrient withdrawals [49]. Harvesting trees during the dormant season or using a defoliant would ensure the tree farm realizes the environmental benefits of the leafy material.

## Conclusions

Growing poplar using a SRC system requires the trees to be harvested after 2-year growth such that the root system is well established. Bioconversion of WTC composed of (whitewood) chip, bark, branch, and leaf from this initial harvest were evaluated and compared to bioconversion of WWF. It was found that leafy material makes up more than a thrid of WTC but has a low sugar content, and high phenolic, ash, and extractives contents. Leaf removal significantly changed the chemical composition of coppice sample, and improved monomeric sugar yield by 147 kg/tonne after steam pretreatment and enzymatic hydrolysis. With the presence of bark and branch, the NLC achieved 67% monomeric sugar recovery, showing no significant difference compared to that of WWF (71%). The overall sugar yield of NLC was 135 kg/tonne less than that of WWF, however, due to the low inherent sugar content of the NLC. These findings demonstrate that it is essential to remove the leaves prior to pretreatment to ensure a better overall sugar yield. A minimum ethanol selling price to cover operating expenses of $1.69/gallon was established from the economic analysis, assuming the NLC feedstock is available for its fuel value.

The growth and maturity of the wood are limited in the first cycle. We would anticipate that the following rotations will have more wood chips, less bark/branch (the second cycle is showing 60% whitewood content in recent harvest), and therefore a higher overall sugar content. In addition, several practices could potentially enhance the compositional characteristics of short rotation poplar and fortify the sugar content, such as choosing higher sugar composition hybrid/clone, modifying the management strategy, and/or mixing with other sugar-rich feedstocks.

## Additional file

**Additional file 1: Table S1.** Relevant process yields and assumptions for economic assessment.

## Abbreviations

SRC: short rotation coppice
SRF: short rotation forestry
WTC: whole tree coppice
NLC: no-leaf coppice
LC: leaf coppice
WWF: whitewood forestry
WSF: water-soluble fraction
WIF: water-insoluble fraction
OD: oven dry
HMF: 5-hydroxymethyl furfural
FPU: filter paper unit
CBU: cellobiase unit
HPLC: high-pressure liquid chromatography
NREL: National Renewable Energy Laboratory
ANOVA: analysis of variance

## References

[1] Humbird D, Davis R, Tao L, Kinchin C, Hsu D, Aden A, et al. Process design and economics for biochemical conversion of lignocellulosic biomass to ethanol. Golden; 2011.

[2] Juneja A, Kumar D, Murthy GS. Economic feasibility and environmental life cycle assessment of ethanol production from lignocellulosic feedstock in Pacific Northwest US. J Renew Sustain Energy. 2013;5:023142. http://scitation.aip.org/content/aip/journal/jrse/5/2/10.1063/1.4803747.

[3] Sannigrahi P, Ragauskas AJ, Tuskan GA (2010). Poplar as a feedstock for biofuels: a review of compositional characteristics. Biofuels Bioprod Biorefin.

[4] Dillen SY, Djomo SN, Al Afas N, Vanbeveren S, Ceulemans R (2013). Biomass yield and energy balance of a short-rotation poplar coppice with multiple clones on degraded land during 16 years. Biomass Bioenergy.

[5] Tuskan GA, Difazio S, Jansson S, Bohlmann J, Grigoriev I, Hellsten U, et al. The genome of black cottonwood, *Populus trichocarpa* (Torr. & Gray). Science. 2006;313:1596–604. http://science.sciencemag.org/content/313/5793/1596.abstract. 10.1126/science.1128691 16973872

[6] Kenney KL, Smith WA, Gresham GL, Westover TL. Understanding biomass feedstock variability. Biofuels. 2013;4:111–27. http://www.scopus.com/inward/record.url?eid=2-s2.0-84871571363&partnerID=tZOtx3y1.

[7] Sims RE, Venturi P (2004). All-year-round harvesting of short rotation coppice eucalyptus compared with the delivered costs of biomass from more conventional short season, harvesting systems. Biomass Bioenergy.

[8] Isebrands J, Karnosky D. Environmental benefits of poplar culture. In: Dickmann DI, Isebrands JG, Eckenwalder JE, Richardson J, editors. Poplar culture in North America. Ottawa: NRC Research Press; 2001. p. 207–218. http://www.nrcresearchpress.com/doi/pdf/10.1139/9780660181455#page=224.

[9] Kauter D, Lewandowski I, Claupein W. Quantity and quality of harvestable biomass from *Populus* short rotation coppice for solid fuel use—a review of the physiological basis and management influences. Biomass Bioenergy. 2003;24:411–27. http://www.sciencedirect.com/science/article/pii/S0961953402001770.

[10] Ceulemans R, Deraedt W. Production physiology and growth potential of poplars under short-rotation forestry culture. For Ecol Manag. 1999;121:9–23. http://www.sciencedirect.com/science/article/pii/S0378112798005647.

[11] Stanton B, Eaton J, Johnson J, Rice D, Schuette B, Moser B (2002). Hybrid poplar in the Pacific Northwest: the Effects of market-driven management. J For.

[12] Njakou Djomo S, Ac A, Zenone T, De Groote T, Bergante S, Facciotto G, et al. Energy performances of intensive and extensive short rotation cropping systems for woody biomass production in the EU. Renew Sustain Energy Rev. 2015;41:845–54. http://www.sciencedirect.com/science/article/pii/S1364032114007345.

[13] Zsuffa L, Giordano E, Pryor LD, Stettler RF. Trends in poplar culture: some global and regional perspective. In: Stettler RF, Bradshaw HD, Heilman PE, Hinckley TM, editors. Biology of *Populas* its implications for management and conservation. Ottawa: NRC Research Press; 1996. p. 515–39. https://books.google.com/books?id=HvuTJC32C3YC&printsec=frontcover#v=snippet&q=coppice&f=false.

[14] Stanturf JA, Van Oosten C, Netzer DA, Coleman MD, Portwood CJ. Ecology and silviculture of poplar plantations. In: Poplar culture North America. Ottawa: NRC Research Press; 2001. p. 153–206.

[15] Hansen EA. Poplar woody biomass yields: a look to the future. Biomass and Bioenergy. 1991;1:1–7. http://www.sciencedirect.com/science/article/pii/096195349190046F.

[16] Tao G, Lestander TA, Geladi P, Xiong S. Biomass properties in association with plant species and assortments I: a synthesis based on literature data of energy properties. Renew Sustain Energy Rev. 2012;16:3481–506. http://www.sciencedirect.com/science/article/pii/S1364032112001335.

[17] Wood Resources International. North American wood fiber review; 2012.

[18] Al Afas N, Marron N, Zavalloni C, Ceulemans R. Growth and production of a short-rotation coppice culture of poplar—IV: fine root characteristics of five poplar clones. Biomass Bioenergy. 2008;32:494–502. http://www.sciencedirect.com/science/article/pii/S0961953407002073.

[19] Mitchell CP. Ecophysiology of short rotation forest crops. Biomass Bioenergy. 1992;2:25–37. http://www.sciencedirect.com/science/article/pii/0961953492900855.

[20] Karp A, Shield I (2008). Bioenergy from plants and the sustainable yield challenge. New Phytol.

[21] Mitchell C, Stevens E, Watters M. Short-rotation forestry—operations, productivity and costs based on experience gained in the UK. For Ecol Manag. 1999;121:123–36. http://www.sciencedirect.com/science/article/pii/S0378112798005611.

[22] Schweier J, Becker G. New Holland forage harvester’s productivity in short rotation coppice: evaluation of field studies from a German perspective. Int J For Eng. 2012;23:82–8. http://www.tandfonline.com/doi/abs/10.1080/14942119.2012.10739964.

[23] Wyman CE, Dale BE, Elander RT, Holtzapple M, Ladisch MR, Lee YY (2009). Comparative sugar recovery and fermentation data following pretreatment of poplar wood by leading technologies. Biotechnol Prog.

[24] DeMartini JD, Wyman CE. Changes in composition and sugar release across the annual rings of *Populus* wood and implications on recalcitrance. Bioresour Technol. 2011;102:1352–8. http://www.sciencedirect.com/science/article/pii/S0960852410015087. 10.1016/j.biortech.2010.08.123 20943384

[25] Schütt F, Puls J, Saake B. Optimization of steam pretreatment conditions for enzymatic hydrolysis of poplar wood. Holzforschung. 2011;65:453–9. http://www.degruyter.com/view/j/hfsg.2011.65.issue-4/hf.2011.066/hf.2011.066.xml.

[26] Sluiter A, Hames B, Ruiz R, Scarlata C, Sluiter J, Templeton D. Determination of ash in biomass: laboratory analytical procedure (LAP); 2008.

[27] Sluiter A, Ruiz R, Scarlata C, Sluiter J, Templeton D. Determination of extractives in biomass: laboratory analytical procedure (LAP); 2008.

[28] Sluiter A, Hames B, Ruiz R, Scarlata C. Determination of sugars, byproducts, and degradation products in liquid fraction process samples (LAP); 2006. http://www.nrel.gov/docs/gen/fy08/42623.pdf.

[29] Dou C, Ewanick S, Bura R, Gustafson R. Post-treatment mechanical refining as a method to improve overall sugar recovery of steam pretreated hybrid poplar. Bioresour Technol. 2016;207:157–65. http://www.sciencedirect.com/science/article/pii/S0960852416300529. 10.1016/j.biortech.2016.01.076 26881333

[30] Singleton VL, Orthofer R, Lamuela-Raventós RM (1999). Analysis of total phenols and other oxidation substrates and antioxidants by means of folin-ciocalteu reagent. Methods Enzymol.

[31] TAPPI Test Methods. Acid-insoluble lignin in wood and pulp, Test Method T 222 om-11. Atlanta; 2001. http://www.tappi.org/Bookstore/Standards-TIPs/Standards/Fibrous-Materials/Acid-Insoluble-Lignin-in-Wood-and-Pulp-Test-Method-T-222-om-06.aspx.

[32] Sluiter A, Hames B, Ruiz R, Scarlata C, Sluiter J, Templeton D, et al. Determination of structural carbohydrates and lignin in biomass: laboratory analytical procedure (LAP); 2008.

[33] Arnold T, Appel H, Patel V, Stocum E, Kavalier A, Schultz J (2004). Carbohydrate translocation determines the phenolic content of *Populus* foliage: a test of the sink-source model of plant defense. New Phytol.

[34] Öhgren K, Bura R, Saddler J, Zacchi G. Effect of hemicellulose and lignin removal on enzymatic hydrolysis of steam pretreated corn stover. Bioresour Technol. 2007;98:2503–10. http://www.sciencedirect.com/science/article/pii/S0960852406004573. 10.1016/j.biortech.2006.09.003 17113771

[35] NASDAQ. Natural gas price: chart for natural gas. 2016. http://www.nasdaq.com/markets/natural-gas.aspx.

[36] Guidi W, Tozzini C, Bonari E (2009). Estimation of chemical traits in poplar short-rotation coppice at stand level. Biomass Bioenergy.

[37] Sjostrom E (1993). Wood chemistry: fundamentals and applications.

[38] Blankenhorn PR, Bowersox TW, Kuklewski KM, Stimely GL. Effects of rotation, site, and clone on the chemical composition of *Populus* hybrids. Wood Fiber Sci. 1985: 351–60. http://wfs.swst.org/index.php/wfs/article/view/730.

[39] Yeh T-F, Chang M-J, Chang W-J (2014). Comparison of dilute acid and sulfite pretreatments on *Acacia confusa* for biofuel application and the influence of its extractives. J Agric Food Chem.

[40] Bin Y, Hongzhang C (2010). Effect of the ash on enzymatic hydrolysis of steam-exploded rice straw. Bioresour Technol.

[41] Ximenes E, Kim Y, Mosier N, Dien B, Ladisch M. Inhibition of cellulases by phenols. Enzyme Microb Technol. 2010;46:170–6. http://www.sciencedirect.com/science/article/pii/S0141022909002543. 10.1016/j.enzmictec.2010.09.006 22112771

[42] Ximenes E, Kim Y, Mosier N, Dien B, Ladisch M. Deactivation of cellulases by phenols. Enzyme Microb Technol. 2011;48:54–60. http://www.sciencedirect.com/science/article/pii/S0141022910001997. 10.1016/j.enzmictec.2010.09.006 22112771

[43] Vera RM, Bura R, Gustafson R. Synergistic effects of mixing hybrid poplar and wheat straw biomass for bioconversion processes. Biotechnol Biofuels. 2015;8:226. http://biotechnologyforbiofuels.biomedcentral.com/articles/10.1186/s13068-015-0414-9. 10.1186/s13068-015-0414-9 PMC4690274 26705420

[44] Thompson VS, Lacey JA, Hartley D, Jindra MA, Aston JE, Thompson DN (2016). Application of air classification and formulation to manage feedstock cost, quality and availability for bioenergy. Fuel.

[45] Devappa RK, Rakshit SK, Dekker RFH. Forest biorefinery: potential of poplar phytochemicals as value-added co-products. Biotechnol Adv. 2015;33:681–716. http://www.sciencedirect.com/science/article/pii/S0734975015000373. 10.1016/j.biotechadv.2015.02.012 25733011

[46] Leng RA, Fujita T. Tree foliage in ruminant nutrition. FAO; 1997. http://www.fao.org/docrep/003/w7448e/w7448e00.htm.

[47] Bals BD, Dale BE, Balan V, Bergeron C, Carrier DJ, Ramaswamy S (2012). Recovery of leaf protein for animal feed and high-value uses. Biorefin Co Prod.

[48] Young RA, Giese RL (1990). Introduction to forest science.

[49] Kimmins JP. Evaluation of the consequences for future tree productivity of the loss of nutrients in whole-tree harvesting. For Ecol Manag. 1976;1:169–83. http://linkinghub.elsevier.com/retrieve/pii/0378112776900190.

