# Supplementary material for: Can we use short rotation coppice poplar for sugar based biorefinery feedstock? Bioconversion of 2-year-old poplar grown as short rotation coppice
Source: Biotechnol Biofuels. 2017 Jun 5;10:144. doi: 10.1186/s13068-017-0829-6 (PMC5460468; doi:10.1186/s13068-017-0829-6)
Supplement: Supplementary file 1 — Additional file 1: Table S1. Relevant process yields and assumptions for economic assessment. [file 13068_2017_829_MOESM1_ESM.docx]

**Can we use short rotation coppice poplar for sugar based biorefinery feedstock? Bioconversion of 2-year-old poplar grown as short rotation coppice**

Chang Dou^1^, Wilian F. Marcondes^1,2^, Jessica E. Djaja^1^, Renata Bura*^1^, and Rick Gustafson^1^

^1^ Biofuels and Bioproducts Laboratory, School of Environmental and Forest Sciences, University of Washington, Seattle, WA 98115, USA

^2^ Department of Biotechnology, Lorena School of Engineering, University of São Paulo, Lorena - Brazil

**Additional Table S1.** Relevant process yields and assumptions for economic assessment

| **Process** | **Parameter** |  |
| --- | --- | --- |
| Feedstock supply | Feedstock price | $53/tonne ^1^ |
| Pretreatment (steam explosion) | Solid recovery | 34% ^2^ |
| Enzymatic hydrolysis | Cellulose to glucose | 72.7% ^2^ |
|  | Xylan to xylose | 54.7% ^2^ |
| Fermentation | Glucose to ethanol | 95% ^3^ |
|  | Xylose to ethanol | 85% ^3^ |
|  | Arabinose to ethanol | 85% ^3^ |
|  | Galactose to ethanol | 0% ^3^ |
|  | Mannose to ethanol | 0% ^3^ |
|  | Beer ethanol concentration | 4.0% ^4^ |
| Utilities | Electricity price | $0.07/kWh |
| Sale | Ethanol price | $1.65/gallon ^5^ |

^1^ The feedstock price was determined based on the heating value of $2.8/MMBtu ($0.00265/MJ)

^2^ Based on current research (experimental data)

^3^ Based on NREL report (2011 biochemical conversion of lignocellulosic ethanol report)

^4^ Based on process simulation (Aspen model)

^5^ Ethanol price based on market price of denatured fuel ethanol (Trading economics, April 17, 2017)

* The operating cost is updated to 2015 USD using the Inorganic Chemical Index and the Labor Index
